# Surgical management of camel-related craniofacial injuries

**DOI:** 10.4314/ahs.v22i3.44

**Published:** 2022-09

**Authors:** Korana Balac, Mohamed A Al-Ali, Ashraf F Hefny, Baraa K Mohamed, Fikri M Abu-Zidan

**Affiliations:** 1 Department of Surgery, Al Ain Hospital, Al Ain, Abu Dhabi, United Arab Emirates; 2 Department of Otolaryngology, Al Ain Hospital, Al Ain, Abu Dhabi, United Arab Emirates; 3 Department of Surgery, College of Medicine and Health Sciences, UAE University, Al Ain, Abu Dhabi, United Arab Emirates; 4 Department of Surgery, Cleveland Clinic Abu Dhabi, Abu Dhabi, United Arab Emirates; 5 The Research Office, College of Medicine and Health Sciences, United Arab Emirates University, Al Ain, Abu Dhabi, United Arab Emirates

**Keywords:** Camel, face injury, head injury, surgery

## Abstract

**Background:**

There are no studies focused on the types and management of camel-related craniofacial injuries.

**Objectives:**

We aimed to analyze the pattern of injuries that required surgical management and their specific operative treatment.

**Methods:**

We prospectively collected data of all patients who were admitted to Al Ain Hospital with camel-related craniofacial injuries that were treated operatively during the period of January 2015 to January 2020.

**Results:**

Eleven patients were studied; all were males having a median (range) age of 29 (19–66) years. Falling from a camel was the most common mechanism of injury (45.5 %) followed by camel bite (36.4 %). The most common injured region was the middle third of the face, which accounted for 56.5% of the bony fractures. Zygomatico-maxillary complex fractures were present in 60% of patients who fell while riding a camel. The most common surgical procedure performed in our patients was an open reduction with internal fixation (54.5%). There was no mortality.

**Conclusions:**

camel-related craniofacial injuries are complex. The main mechanism of injury is falling from a camel on the face causing fractures of the zygomatico-maxillary complex. These fractures usually need open reduction with internal fixation. Taking safety precautions may help in injury prevention.

## Background

Injury to the craniofacial (CF) region is commonly encountered among trauma patients. Due to its proximity to vital organs, these injuries need special attention. They present one of the most difficult challenges for trauma surgeons, as they are often associated with high morbidity, cosmetic disfigurement, as well as functional deficit^1^–^3^. Camels are domestic animals in nearly the entire world. They are commonly found in northern Africa and southwestern Asia. It is estimated that greater than 80% of the world's camel population is in Africa, mostly living in Africa's horn^4^. In Dubai, there are more than 300,000 registered camels^5^. They are considered an important source of food and milk. Furthermore, camel racing is a traditional sport in the United Arab Emirates (UAE). More than 80% of animal-related injuries in the UAE are caused by camels^6^. Contrary to a common belief, injuries caused by camels can be different from those caused by other large animals. Given the camel's size, force, and unpredicted behavior, injuries caused by camels can be life-threatening. The mechanism of camel-related injuries varies and includes kicks, falls, bites, and collisions with vehicles^7^,^8^.

The CF area sustains more frequent and severe injuries including soft tissue lacerations, facial bone fractures, skull fractures, intracranial hemorrhages, and brain injuries^9^–^11^. Injured patients usually require hospital admission for operative management^12^,^13^.

In contrast to human injuries caused by other large animals, camel-related injuries have attracted scant attention in the literature. To our knowledge, no studies have focused on the types and surgical management of camel-related CF injuries. In this study, we aimed to analyze the pattern of camel-related CF injuries that required surgical management and types of operations which were performed.

## Methods

### Ethical considerations

Ethical approval for this study was obtained from AAH Research Ethics Governance Committee, Al Ain Hospital, Al Ain, Abu Dhabi, UAE (AAHEC-04-18-086). Written informed consent was taken from the patients who agreed to publish their clinical data.

### Data collection

We prospectively collected data of all patients who were admitted to Al Ain Hospital with camel-related CF injuries that were treated operatively during the period of January 2015 to January 2020. Al-Ain Hospital is a university-affiliated community-based hospital having trauma and acute care facilities. It is located in Al Ain City, which has a population of 738,000 inhabitants^14^.

A special study protocol was designed to study the details of craniofacial injuries. Data collected included demography, vital signs, and Glasgow Coma Score (GCS) on admission, mechanism of injury, anatomical location and severity of the injury, associated injuries, surgical management. The patients were followed up during their hospital stay to record the length of hospital stay (LOS), complications, and outcome. All CF fractures were classified and confirmed by preoperative imaging and operative findings. Patients with facial fractures were classified depending on the affected region of the face into upper third, middle third, and lower third. The severity of the injury of an anatomical region was assessed by the Abbreviated Injury Severity Score (AIS).

Overall injury severity was determined using the Injury Severity Score (ISS). Both were calculated manually using the AIS 2008 handbook^15^.

### Statistical analysis

The collected data were entered into a Microsoft Excel spreadsheet (Microsoft Corporation, Seattle, WA). A simple descriptive statistical analysis was performed. Data were presented as median (range) or number (%) as appropriate. Statistical analyses were performed using the Statistical Package for the Social Sciences (IBM-SPSS version 26, Chicago, Il).

## Results

Eleven patients were studied; all were males. Their median (range) age was 29 (19–66) years.

The median (range) ISS was 4 (1–16), and the median (range) GCS was 15 (10–15). Ten patients (91%) were from the Indian subcontinent. None of our patients had used a helmet or any other protective gear at the time of injury. Two patients (18.2%) had an injury to another body region besides the CF complex; one had a neck injury, and the other had an upper limp injury.

The primary mechanism of injury was fall while riding a camel in five patients (45.5 %), camel bite in four patients (36.4 %), and camel kick to the face in two patients (18.2 %). A secondary mechanism of injury occurred in four patients who were stepped on by a camel.

**Figure 1:**
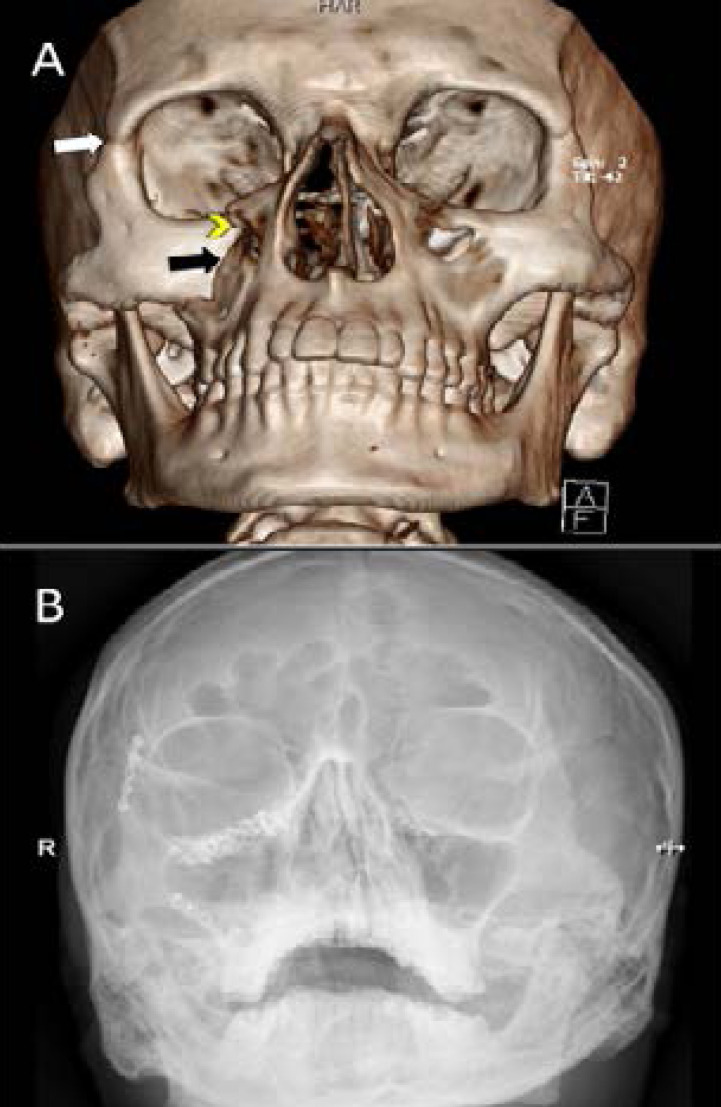
A 31-year-old camel caregiver was kicked by a camel into the face. A three-dimensional (3D) CT scan (A) demonstrates a depressed fracture of the right zygomatic bone (black arrow), fracture of the right lateral orbital wall (white arrow), and orbital floor (yellow arrowhead). Postoperative plain radiograph (B) showing reconstruction of facial fractures with open reduction and internal fixation.

**Figure 2:**
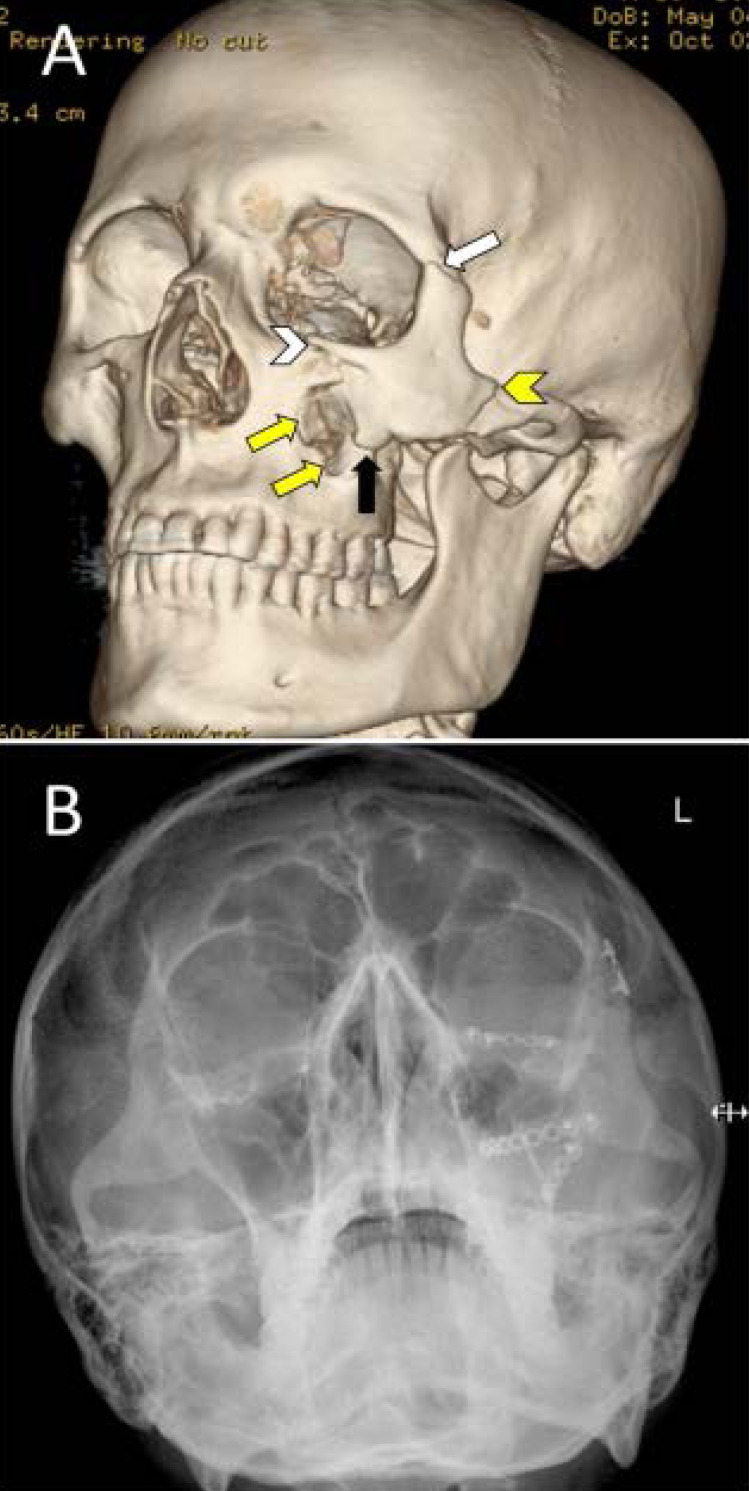
A 30- year old camel caregiver fell off while riding a camel followed by being stepped on his left side of the face by another camel. A three-dimensional (3D) CT scan (A) demonstrates ZMC fracture with disruption of the left zygomaticofrontal suture (white arrow), the zygomaticomaxillary suture (black arrow), and the zygomaticotemporal suture (yellow arrowhead). Note also the displaced fracture of the left orbital floor (white arrowhead) and the anterior wall of the maxillary sinus (yellow arrows). Postoperative plain radiograph (B) showing reconstructed facial fractures with open reduction and internal fixation.

**Figure 3:**
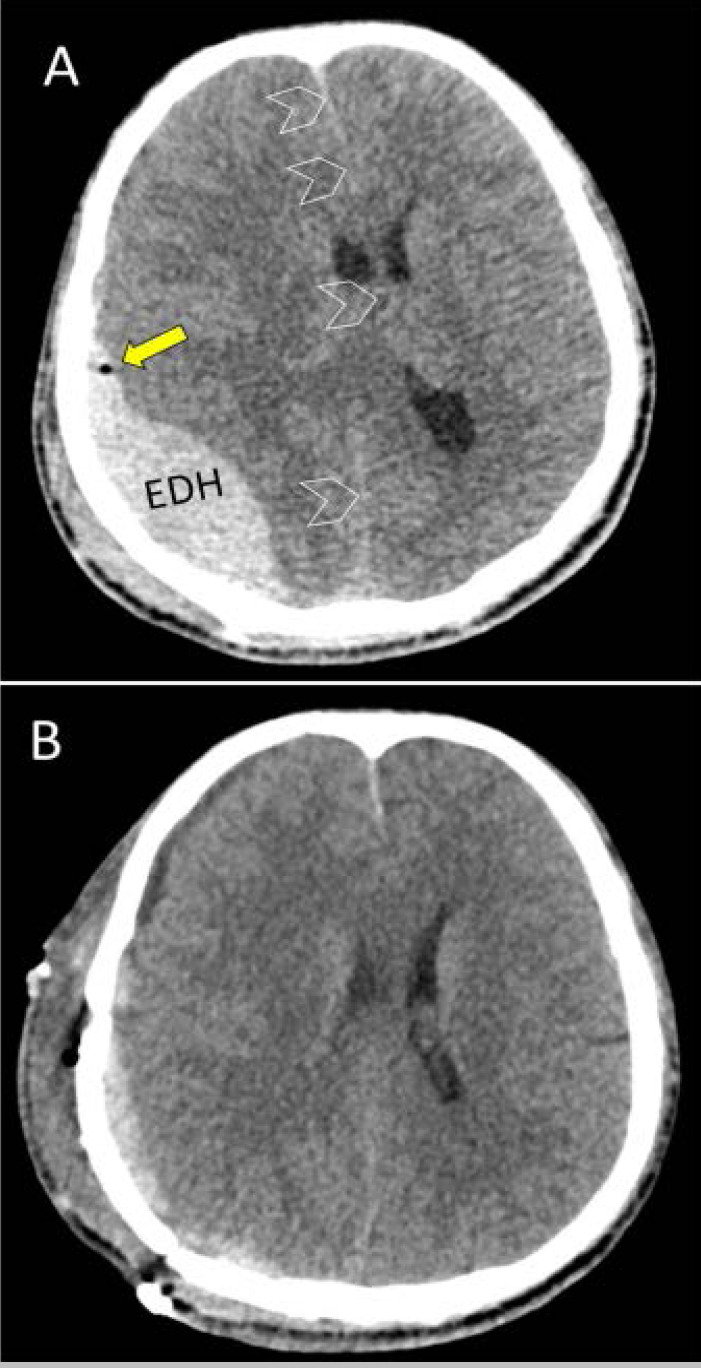
A 29-year-old camel caregiver fell off a camel, followed by being stepped on his head by the camel's hoof. Axial CT scan (A) of the head demonstrates right-sided parieto-occipital epidural hematoma (EDH) with midline shift (arrowheads) associated with intracranial air (yellow arrow) and hematoma of the right scalp. Postoperative day one CT scan (B) following right craniotomy showing a significant reduction of EDH.

The distribution of CF fractures by anatomical sites is shown in Table 1. The most common injured region was the middle third of the face, which accounted for 56.5% of the bony fractures. Maxillary bone and orbital bone fractures were the most frequent mid-facial fractures (Figure 1). Zygomatico-maxillary complex (ZMC) fractures were present in three out of the five patients who fell while riding a camel (Figure 2). Mandibular fractures were observed in two patients who were kicked by a camel. Two patients had an isolated skull fracture, of which one had a concomitant intracranial hemorrhage with a midline shift (Figure 3).

**Table 1:** Fracture subunit distribution in 11 patients with craniofacial camel-related injuries during the period of 2015–2020, Al Ain Hospital, Al Ain, Abu Dhabi, UAE

Fracture	No (%) of patients (n=11)	No. (%) of fractures (n=23)
Skull	2 (18%)	4 (17.4%)
Upper face:	1 (9%)	1 (4.3%)
Frontal bone	1 (9%)	1 (4.3%)
Middle face:	6 (54.5%)	13 (56.5%)
Maxilla	4 (36.3%)	4 (17.4%)
Nasal bone	2 (18%)	2 (8.7%)
Orbital	4 (36.3%)	4 (17.4%)
Zygoma	3 (27.2%)	3 (13%)
Lower face:	3 (27.2%)	5 (21.7%)
Mandible	2 (18%)	3 (13%)
Dentoalveolar	2 (18%)	2 (8.7%)

The total number of procedures performed in the 11 patients was 21 (an average of two procedures per patient). The median time to surgery following hospital admission was 12 hours. Specific surgical procedures performed on our patients are shown in Table 2. All operative procedures except one were performed under general anesthesia. Six (54.5%) patients underwent open reduction and internal fixation of fractures (ORIF) (Figure 1), while three (27%) patients underwent closed reduction of facial fractures. Mandibulo-Maxillary fixation (MMF) and arch bars were implanted in two (18%). One patient needed an evacuation of an extradural hematoma (EDH) through a craniotomy (Figure 3).

**Table 2:** Specific surgical procedures performed on 11 patients with craniofacial camel-related injuries during the period of 2015–2020, Al Ain Hospital, Al Ain, Abu Dhabi, UAE

Surgical procedure	No (%) of patients (n=11)	No. (%) of procedures (n=21)
Closed reduction	2 (18%)	3 (14.2%)
ORIF	6 (54.5%)	6 (28.5%)
MMF/ arch bars	2 (18%)	2 (9.5%)
Reconstruction- PDS	2 (18%)	2 (9.5%)
Reconstruction- Ti mesh	1 (9%)	1 (4.7%)
Eye evisceration	1 (9%)	1 (9.5%)
Cranioplasty	1 (9%)	1 (9.5%)
Craniotomy	1 (9%)	1 (9.5%)
Soft tissue laceration repair	4 (36%)	4 (19%)

ORIF: open reduction and internal fixation; MMF: mandibulo-maxillary fixation; PDS: polydioxanone sheet; Ti: titanium

Patients stayed for a median (range) of 4 (1–12) days in the hospital. Two patients were intubated in the Emergency room. One patient (9%) was admitted to the Intensive Care Unit (ICU) after surgery, while the other was successfully extubated after surgery. There was no mortality among our patients. Four patients developed complications, including inferior alveolar nerve paresthesia in two patients and malunion in another. The fourth patient had multiple complications after being repeatedly bitten by a camel to his face, including unilateral vision loss, facial nerve paralysis, and salivary gland fistula.

## Discussion

This study has shown that all patients were young males. This finding is similar to Boffano et al. who analyzed data of 3396 patients with maxillofacial fractures and found that young men were the most frequent victims of maxillofacial trauma^16^. The main mechanism of injury was falling from a camel on the face causing fractures of the ZMC. The most commonly performed surgical procedure was an open reduction with internal fixation (ORIF) of a fracture. In comparison, Weber et al.^17^ showed that emergency surgeries were more common in patients injured by horse kicks.

Most of the camel caregivers in the UAE are low-income workers from the Indian subcontinent who were raising camels in their home country^10^. The majority of their injuries occurred at farms. Moss et al.^18^ showed that the use of safety helmets significantly reduced the severity of horse-related head trauma. None of our patients used a helmet or face shield at the time of injury.

Similar to others^3^,^6^, the middle third of the face was the most frequently injured area in our patients, including maxillary and orbital bones (Table 1, Figure 1). Globally, road traffic crashes remain a major cause of maxillofacial trauma^19^. We noted that ZMC fractures were common in patients who fell from a camel (Figure 2). Most of these patients had an additional secondary mechanism of injury of being stepped on the head and face by a camel's hoof. This has significantly increased the complexity and severity of these fractures. In comparison, orbital fractures were the most common injury associated with horse-related injuries^17^. Camel bites were the second most common injury mechanism in our patients. These bites resulted in severe injuries to the face, including deep lacerations, globe rupture, facial nerve and parotid duct injuries, and facial bone fractures involving orbital and frontal bones. In contrast to horse bites which cause crushing injuries, wounds from camel bites can cause serious punctures and lacerations. Using its sharply pointed canine teeth and strong long jaw, the victim can be efficiently gripped, lifted, and shaken with a jerky movement. Deep body structures can be severely injured despite the superficial appearance of the wound^7^,^10^. Two patients were kicked by a camel to their jaw resulting in mandibular fractures. This occurred because of the direction and high energy of the camel kick^3^,^6^. Ruslin et al. found a higher risk of dental injury among patients with mandibular fractures. Similarly, patients with mandibular fractures in our series had associated dentoalveolar fractures^20^.

CF injuries can significantly alter the structure, function, and appearance of the injured site. Therefore, the decision to operate on these patients should be taken early. In contrast to other studies^16^,^21^,^22^, the median time to surgery in our patients was much shorter, 12 hours after hospital admission. Boffano et al.^16^, showed that 45 % of maxillofacial fractures were treated after 72 hours of injury, and only 37 % were managed within 24 hours. Patients with camel-related CF trauma may require more than one surgical procedure. In our study, an average of two operative procedures per patient was required. Most of our patients had multiple CF injuries involving soft tissue and bony structures. Over half of our patients were managed with ORIF using titanium mini-plates (Table 2). Using this technique, one can optimally restore function and achieve pre-trauma occlusion and esthetics. MMF with arch bars was used in two of our patients who had dentoalveolar and mandibular fractures (Table 2). Patients with orbital floor fractures were reconstructed with titanium mesh and polydioxanone sheet (PDS). In contrast to others^6^, the overall outcome of our patients was good, with no reported mortality. More than ninety percent of patients were discharged in good condition and without permanent disabilities. We think this is because we have managed our patients early, and most had minor head and concomitant injuries.

We acknowledge that our sample size is small. Nevertheless, this manuscript is unique in its nature, given the scarcity of these injuries. Furthermore, the details of the surgical management of camel-related CF injuries were not studied before.

## Conclusion

Camel-related CF injuries are challenging and complex. The main mechanism of injury is falling from a camel on the face causing fractures of the zygomatico-maxillary complex. These fractures are managed with open reduction with internal fixation. Taking safety precautions when handling camels may help in injury prevention.

## References

[1] Bresler AY, Hanba C, Svider P, Carron MA, Hsueh WD, Paskhover B (2019). Craniofacial injuries related to motorized scooter use: A rising epidemic. American journal of otolaryngology.

[2] Arslan ED, Solakoglu AG, Komut E, Kavalci C, Yilmaz F, Karakilic E, Durdu T, Sonmez M (2014). Assessment of maxillofacial trauma in emergency department. World Journal of Emergency Surgery.

[3] Al-Ali MA, Hefny AF, Abu-Zidan FM (2019). Head, face and neck camel-related injuries: Biomechanics and severity. Injury.

[4] Faye B (2014). The Camel today: assets and potentials. Anthropozoologica.

[5] Chaudhary S (2018). Understanding the Emirati love for camels. Gulf News.

[6] Abu-Zidan FM, Hefny AF, Eid HO, Bashir MO, Branicki FJ (2012). Camel-related injuries: prospective study of 212 patients. World Journal of Surgery.

[7] Balac K, Al-Ali MA, AlMahmoud T, Abu-Zidan FM (2019). Globe rupture caused by a camel bite. Trauma Case Report.

[8] Eid HO, Hefny AF, Abu-Zidan FM (2015). Epidemiology of animal-related injuries in a high-income developing country. Ulusal travma ve acil cerrahi dergisi. Turkish journal of trauma & emergency surgery : TJTES.

[9] Ugboko VI, Olasoji HO, Ajike SO, Amole AO, Ogundipe OT (2002). Facial injuries caused by animals in northern Nigeria. The British journal of oral & maxillofacial surgery.

[10] Kain R, Arya S (2015). Camel bite: An uncommon mode of maxillofacial injury, its mechanism and fatality: Case series and review of literature. National Journal of Maxillofacial Surgery.

[11] Islam S, Gupta B, Taylor CJ, Chow J, Hoffman GR (2014). Equine-associated maxillofacial injuries: retrospective 5-year analysis. The British journal of oral & maxillofacial surgery.

[12] Singleton C, Manchella S, Nastri A (2019). Operative management of equine-related maxillofacial trauma presenting to a Melbourne level-one trauma centre over a six-year period. The British journal of oral & maxillofacial surgery.

[13] Dobitsch AA, Oleck NC, Liu FC, Halsey JN, Hoppe IC, Lee ES (2019). Sports-Related Pediatric Facial Trauma: Analysis of Facial Fracture Pattern and Concomitant Injuries. Surgery journal (New York, NY).

[14] Statistic Centre AbuDhabi (2016). Statistical yearbook population.

[15] Gennarelli TA, Wodzin E (2008). Abbreviated injury scale 2005 update. Association for the Advancement of Automotive Medicine.

[16] Boffano P, Roccia F, Zavattero E, Dediol E, Uglešić V, Kovačič Ž (2015). European Maxillofacial Trauma (EURMAT) project: a multicentre and prospective study. J Craniomaxillofac Surg.

[17] Weber CD, Nguyen AR, Lefering R, Hofman M, Hildebrand F, Pape HC (2017). Blunt injuries related to equestrian sports: results from an international prospective trauma database analysis. Int Orthop.

[18] Moss PS, Wan A, Whitlock MR (2002). A changing pattern of injuries to horse riders. Emergency Medicine Journal.

[19] Boffano P, Kommers SC, Karagozoglu KH, Forouzanfar T (2014). Aetiology of maxillofacial fractures: a review of published studies during the last 30 years. Br J Oral Maxillofac Surg.

[20] Ruslin M, Wolff J, Boffano P, Brand HS, Forouzanfar T (2015). Dental trauma in association with maxillofacial fractures: an epidemiological study. Dent Traumatol.

[21] Brucoli M, Boffano P, Romeo I, Corio C, Benech A, Ruslin M (2020). Management of maxillofacial trauma in the elderly: A European multicenter study. Dental traumatology: official publication of International Association for Dental Traumatology.

[22] Shew M, Carlisle MP, Lu GN, Humphrey C, Kriet JD (2016). Surgical Treatment of Orbital Blowout Fractures: Complications and Postoperative Care Patterns. Craniomaxillofacial trauma & reconstruction.

